# Prenatal and Childhood Growth, Chemerin Concentrations, and Metabolic Health in Adult Life

**DOI:** 10.1155/2016/3838646

**Published:** 2016-01-24

**Authors:** Johan G. Eriksson, Mika Venojärvi, Clive Osmond

**Affiliations:** ^1^National Institute for Health and Welfare, Department of Chronic Disease Prevention, 00271 Helsinki, Finland; ^2^Department of General Practice and Primary Health Care, University of Helsinki and Helsinki University Hospital, 00014 Helsinki, Finland; ^3^Folkhälsan Research Centre, 00250 Helsinki, Finland; ^4^Institute of Biomedicine, Exercise Medicine, University of Eastern Finland, 70211 Kuopio, Finland; ^5^MRC Lifecourse Epidemiology Unit (University of Southampton), Southampton General Hospital, Southampton SO16 6YD, UK

## Abstract

Several noncommunicable diseases have their origins in early developmental phases. One factor possibly explaining the association between early growth and later health could be adipocyte function. The objective of this study was to assess the association between the adipocytokine chemerin and early growth and later health. 1074 participants from Helsinki Birth Cohort Study born 1934–1944 with information on prenatal and childhood growth participated. Metabolic outcomes include glucose tolerance, adiposity, and chemerin concentration. Mean chemerin concentrations were 5.0 ng/mL higher in women than in men (95% CI 2.7 to 7.2, *p* < 0.001). The strongest correlate of chemerin concentration was adult waist circumference and body fat percentage (*r* = 0.22, *p* < 0.001 and *r* = 0.21, *p* < 0.001, resp.). After adjustment for body fat percentage, chemerin concentration was 5.4 ng/mL lower in subjects with type 2 diabetes than in those with normal glucose tolerance (−0.2 to 10.9, *p* = 0.06). It was 3.0 ng/mL higher in those with metabolic syndrome than in those without (0.6 to 5.3, *p* = 0.01). No measure of early growth was associated with chemerin concentration. Our findings do not support a role for chemerin in linking early growth with later metabolic health.

## 1. Introduction

Several noncommunicable diseases, including type 2 diabetes (T2D) and coronary heart disease (CHD), have their origins in early developmental phases [1]. The intrauterine environment is largely influenced by maternal diet, maternal body size, placental function, and hormonal factors that can affect the fetus via several mechanisms causing life-long programming of health. Not only prenatal experiences but also growth and experiences during childhood can have long-term health consequences [2–4]. We and others have shown that T2D and CHD are associated with body size at birth but even more strongly with a mismatch between body size at birth and body size later in childhood [5, 6].

One possible factor explaining the association between early growth and later health outcomes could be adipocyte function and adipocytokine production. Chemerin is a recently identified adipocytokine shown to be associated with components of the metabolic syndrome [7–12]. Strong correlations have been described between chemerin and several metabolic outcomes including obesity and metabolic syndrome. Due to the close association between early growth and these metabolic outcomes chemerin could be one common denominator explaining these associations. To our knowledge no previous study has assessed the association between chemerin concentration and prenatal and childhood growth and later metabolic health, which was the primary aim of the present study.

## 2. Subjects and Methods

The Helsinki Birth Cohort Study (HBCS) comprises 13345 men and women who were born in one of the two maternity hospitals in the city between 1934 and 1944 and who attended child welfare clinics in the city. These clinics were free of charge and almost all children attended them. The majority of the children also went to school in the city and had growth data from school health records available. Details of the records at birth, in the clinics and at school, have been described [3, 6]. Each subject had an average of 11 measurements of height and weight from birth to 2 years of age and 6 measurements of height and weight from 2 to 11 years. Weight and height at age of 20 years were obtained from questionnaire.

We used random number tables to select a subsample of people who were still alive and living in Finland in the year 2000 for a clinical study in the years 2001–2003. In order to achieve a sample size in excess of 2000 people for this subset we selected 2691 subjects for evaluation. Of these subjects, 2003 attended a clinical study after an overnight fast. A follow-up study including 1083 participants was conducted in the years 2006–2008, focusing upon individuals who were without manifest type 2 diabetes at the time of the original study (2001–2003). The exclusion of people with known diabetes was because one of the study aims was to focus upon development of diabetes during a 5-year follow-up period in an elderly group of people. Of these 1074 who had chemerin concentration measured were included in this study.

Height and weight were measured in light indoor clothing and without shoes with a KaWi stadiometer (nearest 0.1 cm) and a SECA alpha 770 scale (nearest 0.1 kg), respectively. Body composition was assessed by bioimpedance using the In Body 3.0 device. Blood pressure was measured from the right arm while the subject was in the sitting position, and it was recorded as the mean of 2 successive readings from a mercury sphygmomanometer. A 2-hour 75 g oral glucose tolerance test was performed. Type 2 diabetes and impaired glucose tolerance were defined using WHO criteria [13]. Glucose was measured according to the hexokinase method. Plasma insulin concentrations were determined by a two-site immunometric assay [14, 15]. Serum cholesterol and triglyceride concentrations were measured with the use of standard enzymatic methods [16, 17]. LDL-cholesterol concentrations were calculated using the Friedewald formula [18]. Metabolic syndrome was assessed using the IDF criteria [19]. Chemerin was measured from serum samples with Millipore's Human Chemerin Kit (EZHCMRN.57K) using a Thermo Multiskan (Thermo Clinical Labsystems Oy, Konelab, Finland).

### 2.1. Ethical Statement

The study was approved by the Ethics Committee of Hospital District of Helsinki and Uusimaa and conducted according to the guidelines of the Declaration of Helsinki. Written informed consent was obtained from all subjects.

### 2.2. Statistical Methods

We analyzed the data using partial correlation analysis and multiple linear regressions. With clinic variables, we always included age as a predictor. We included sex as a predictor in pooled analyses. Triglyceride and insulin concentrations were right-skewed in distribution and were log-transformed for analysis.

## 3. Results

Summary statistics for the adult characteristics of the 1074 subjects are shown in Table 1. Mean chemerin concentrations were 5.0 ng/mL higher in women than in men (95% confidence interval: 2.7 to 7.2, *p* < 0.001). The strongest correlates of chemerin concentration were adult body fat percentage and waist circumference. Adjusting for body fat percentage explained the association with all other measurements of current body size except that of waist circumference (Table 1). Weak positive associations of chemerin concentration with diastolic blood pressure and fasting glucose concentration were explained by adjustment for body fat percentage. However positive associations with triglyceride and fasting insulin concentrations and a negative association with high density lipoprotein cholesterol concentration remained after such adjustment (Table 1).

Summary statistics for the maternal, neonatal, and childhood characteristics of the subjects are shown in Table 2. No measure of maternal, neonatal, infant, or childhood anthropometry was associated with chemerin concentration, either with or without adjustment for adult body fat percentage (Table 2). Nor was there any association with gestational age at delivery, with head circumference, or with placental weight.

Table 3 shows mean levels of chemerin concentration in men and women according to their glucose tolerance status and the presence of the metabolic syndrome. After adjustment for body fat percentage and pooling across men and women, chemerin concentration was 5.4 ng/mL lower in subjects with type 2 diabetes than in those with normal glucose tolerance (−0.2 to 10.9, *p* = 0.06), and it was 3.0 ng/mL higher in those with metabolic syndrome (IDF criteria) than in those without (0.6 to 5.3, *p* = 0.01).

## 4. Discussion

Several noncommunicable diseases including T2D and CHD have been shown to be associated with markers of early growth [1–6]. Chemerin, a recently identified adipocytokine, has been suggested to be associated with prenatal growth, as well as being a strong marker of insulin resistance and the metabolic syndrome [7–12, 20, 21]. In the present study chemerin was strongly and positively associated with adiposity in adult life especially with body fat percentage and waist circumference. Women had higher chemerin concentrations than men. When adjusting for body fat percentage chemerin concentrations were higher in those with the metabolic syndrome but lower among those with manifest type 2 diabetes. Due to the close association between early growth and several metabolic and adiposity related outcomes chemerin could be one potential denominator explaining these associations. We did not find any association between prenatal or childhood growth and chemerin concentrations in adult life. Therefore our findings do not support a major role for chemerin in linking early growth with later metabolic health.

A recent meta-analysis concluded that chemerin concentrations in obese subjects and in subjects with metabolic syndrome seem to be associated with obesity and disturbances in lipid, glucose, and insulin metabolism [12]. In the present study there was a strong association between different measures of adiposity in adult life and chemerin concentrations. Women had higher chemerin concentrations than men, probably as a consequence of higher body fat percentage. Also, individuals with MS had significantly higher chemerin concentrations. This is consistent with previous studies. There have even been suggestions that chemerin might play a role in the development of obesity and MS [7–12, 22, 23]. Some previous studies, but not all, have reported elevated levels of chemerin in T2D subjects [7, 23]. In the present study we report lower chemerin levels in people with T2D. One reason could be the fact that we were able to adjust for adult body composition. Another reason could be that our study included only recently diagnosed T2D subjects, due to the study design. This might have major impact on the results since some diabetes drugs are known to affect chemerin concentration. Increased levels of chemerin, occurring in association with obesity, have been proposed to be one factor in the development of type 2 diabetes. It has in fact been suggested that chemerin might present either inhibitory or stimulatory effects on glucose uptake through various mechanisms, for example, through glucose-stimulated insulin secretion [24]. In other words published studies on chemerin concentration in type 2 diabetic subjects are inconsistent possibly due to the heterogenous nature of type 2 diabetes, possible impact of diabetes drugs, and disease duration. Only longitudinal studies are able to assess whether the association between the metabolic syndrome and chemerin will reverse when manifest type 2 diabetes develops. Supporting the present findings decreased chemerin levels have also been reported in women with gestational diabetes. It has been suggested that this contributes to the insulin resistance associated with GDM [25]. Elevated chemerin concentrations have also been reported in subjects with CHD, dyslipidemia, and inflammatory states, all closely associated with the metabolic syndrome and T2D [26–31].

Chemerin is expressed in the placenta and also secreted by the placenta emphasizing its role in controlling several metabolic processes during pregnancy. Increased chemerin levels are seen especially during late gestation supporting a role for chemerin in fetal growth [20, 21, 32]. Higher chemerin concentrations have been reported in cord blood from obese women. Consequently higher chemerin concentrations in cord blood samples of large size neonates have also been reported [33]. Maternal serum chemerin levels also differed between preeclamptic and healthy pregnant women, increasing with the severity of preeclampsia [34, 35]. Besides regulating fetomaternal metabolism, a significant increase of chemerin has been observed in polycystic ovary syndrome [36]. We did not find any significant associations between maternal, prenatal, placental, infant, or childhood growth and characteristics and chemerin concentrations in adult life. However, chemerin concentration was strongly associated with adiposity. We have previously shown that prenatal growth is more strongly associated with lean body mass than with fat mass [37]. This could be one reason why no association between early growth and chemerin concentrations was observed.

There are some limitations to our study. Since the follow-up study in 2006–2008 primarily included individuals without diabetes in 2001–2003, we are studying primarily nondiabetic representatives of the age group of 62 to 74 years, including recently diagnosed type 2 diabetic subjects. Our cohort only includes individuals born in Helsinki who attended child welfare clinics in the city and might therefore not be representative of the Finnish population. However, socioeconomic status birth, defined as father's highest attained occupational status, was comparable to the contemporary Helsinki population. All subjects were born and/or grew up during World War II, which might have influenced both nutritional status and growth during childhood. The results, therefore, might not be generalizable to children born and growing up today, but we see no reason why this would influence chemerin concentrations. Finally we do not have information of body composition at birth and during childhood in the elderly population. This is certainly one limitation since it is possible that body composition at birth might have long-term health consequences.

We conclude that chemerin concentrations are strongly associated with adiposity among elderly subjects. Prenatal, placental, infant, and childhood characteristics and growth measurements were not associated with chemerin concentrations. Our findings do not support a major role for chemerin in linking early growth with later metabolic health. Chemerin appears to be a marker of adiposity and body fat mass and this should be taken into account in further studies on chemerin and its function in metabolism.

## Figures and Tables

**Table 1:** Descriptive data for adult clinical measurements and correlations with chemerin concentration.

Adult clinical measurements	Men (*n* = 480)	Women (*n* = 594)	Correlation with chemerin concentration	
	Mean (SD)	Mean (SD)	Direct^‡^	Adjusted^†^
Age (years)	61.5 (2.7)	61.6 (3.0)	—	—
Height (cm)	177.0 (6.0)	163.5 (5.7)	−0.04	0.01
Weight (kg)	84.7 (12.1)	72.4 (12.2)	0.15^*∗∗∗*^	0.01
BMI (kg/m^2^)	27.0 (3.5)	27.1 (4.4)	0.18^*∗∗∗*^	0.01
Waist circumference (cm)	99.0 (10.3)	89.4 (11.3)	0.22^*∗∗∗*^	0.09^*∗∗*^
Lean body mass (kg)	65.0 (7.2)	47.6 (5.4)	0.07^*∗*^	0.03
Fat mass (kg)	19.7 (7.2)	24.5 (8.3)	0.19^*∗∗∗*^	0.00
Body fat percentage (%)	22.8 (5.6)	33.2 (6.4)	0.21^*∗∗∗*^	—
Systolic blood pressure (mmHg)	144.3 (18.5)	142.0 (20.2)	0.03	−0.01
Diastolic blood pressure (mmHg)	89.7 (9.9)	86.8 (9.9)	0.06^*∗*^	0.01
Total cholesterol (mmol/L)	5.8 (1.0)	6.0 (1.0)	0.02	0.01
Triglyceride (mmol/L)^Δ^	1.5 (0.8)	1.4 (0.7)	0.19^*∗∗∗*^	0.13^*∗∗∗*^
HDL cholesterol (mmol/L)	1.5 (0.4)	1.8 (0.4)	−0.15^*∗∗*^	−0.10^*∗∗*^
LDL cholesterol (mmol/L)	3.7 (0.8)	3.7 (0.9)	0.03	0.01
Fasting glucose (mmol/L)	5.7 (0.6)	5.4 (0.6)	0.09^*∗∗*^	0.05
Fasting insulin (pmol/L)^Δ^	10.2 (7.0)	9.2 (10.4)	0.15^*∗∗∗*^	0.07^*∗*^
Chemerin (ng/mL)	66.2 (17.7)	71.2 (19.1)	*1.00*	*1.00*

^‡^Adjusted for age at clinic and sex. ^†^Adjusted for age at clinic, sex, and adult body fat percentage.

^Δ^Variable log-transformed for analysis. ^*∗*^ *p* < 0.05; ^*∗∗*^ *p* < 0.01; ^*∗∗∗*^ *p* < 0.001.

**Table 2:** Descriptive data for maternal, neonatal, and childhood measurements and correlations with chemerin concentration.

Maternal, neonatal, and childhood measurements	Men (*n* = 480)	Women (*n* = 594)	Correlation with chemerin concentration	
	Mean (SD)	Mean (SD)	Direct^‡^	Adjusted^†^
Maternal measurements				
Age (years)	28.8 (5.4)	28.7 (5.4)	−0.01	−0.00
Height (cm)	159.6 (6.0)	159.6 (5.6)	0.02	0.04
Weight (kg)	67.2 (7.6)	67.4 (8.0)	−0.01	−0.01
BMI (kg/m^2^)	26.4 (2.8)	26.4 (2.8)	−0.02	−0.03
Neonatal measurements				
Gestational age (days)	297 (11)	280 (11)	−0.00	−0.01
Birth weight (g)	3485 (480)	3363 (458)	−0.03	−0.02
Birth length (cm)	50.7 (2.0)	50.1 (1.9)	−0.02	−0.00
Head circumference (cm)	35.5 (1.5)	34.9 (1.4)	−0.02	−0.03
Ponderal index (kg/m^3^)	26.7 (2.4)	26.7 (2.2)	−0.02	−0.03
Placental weight (g)	661 (119)	644 (121)	−0.05	−0.05
Childhood measurements				
Age 1 year				
Height (cm)	76.7 (2.5)	75.1 (2.5)	0.01	0.03
Weight (kg)	10.5 (1.0)	9.9 (1.0)	0.02	0.02
BMI (kg/m^2^)	17.9 (1.4)	17.6 (1.4)	0.02	0.01
Age 2 years				
Height (cm)	86.8 (3.0)	85.8 (3.0)	0.01	0.03
Weight (kg)	12.5 (1.1)	12.0 (1.1)	0.01	0.02
BMI (kg/m^2^)	16.7 (1.2)	16.5 (1.2)	0.01	0.00
Age 7 years				
Height (cm)	121.0 (4.8)	120.3 (4.6)	0.01	0.03
Weight (kg)	22.6 (2.6)	22.4 (2.8)	0.02	0.03
BMI (kg/m^2^)	15.5 (1.1)	15.5 (1.3)	0.03	0.01
Age 11 years				
Height (cm)	141.7 (5.7)	141.7 (6.5)	−0.01	0.01
Weight (kg)	33.8 (4.4)	34.3 (5.6)	0.03	0.02
BMI (kg/m^2^)	16.8 (1.4)	17.1 (1.9)	0.05	0.02
Age 20 years				
Height (cm)	177.0 (6.0)	163.5 (5.7)	−0.04	0.01
Weight (kg)	70.9 (8.0)	56.2 (6.8)	0.01	−0.01
BMI (kg/m^2^)	22.6 (2.2)	21.0 (2.4)	0.05	−0.01

^‡^Adjusted for age at clinic and sex. ^†^Adjusted for age at clinic, sex and adult body fat percentage.

No correlations were statistically significant at the 5% level.

**Table 3:** Chemerin concentration according to glucose tolerance or metabolic syndrome in men and women.

	Chemerin concentration (ng/mL)					
	Men			Women		
	*n*	Mean (SD)	*p* value^†^	*n*	Mean (SD)	*p* value^†^
Glucose tolerance^‡^						
Normal	336	66.5 (17.8)	Baseline	409	70.7 (19.5)	Baseline
Impaired	118	66.3 (17.8)	0.4	165	72.7 (18.2)	0.9
Type 2 diabetes	26	62.0 (15.0)	0.1	18	67.0 (15.6)	0.3
Metabolic syndrome^‡^						
Absent	185	63.9 (17.2)	Baseline	335	68.4 (18.0)	Baseline
Present	294	67.5 (17.8)	0.2	259	74.8 (19.8)	0.03
All subjects	480	66.2 (17.7)		594	71.2 (19.1)	

^†^Adjusted for age and body fat percentage and compared to normal glucose tolerance or no metabolic syndrome groups.

^‡^Glucose tolerance not known for two women; metabolic syndrome status not known for one man.
